# Workplace Gaslighting Is Associated with Nurses’ Job Burnout and Turnover Intention in Greece

**DOI:** 10.3390/healthcare13131574

**Published:** 2025-07-01

**Authors:** Ioannis Moisoglou, Aglaia Katsiroumpa, Olympia Konstantakopoulou, Ioanna V. Papathanasiou, Aggeliki Katsapi, Ioanna Prasini, Maria Chatzi, Petros Galanis

**Affiliations:** 1 Department of Nursing, University of Thessaly, 41500 Larissa, Greece; iomoysoglou@uth.gr (I.M.); iopapathanasiou@uth.gr (I.V.P.); mchatzi@uth.gr (M.C.); 2 Clinical Epidemiology Laboratory, Faculty of Nursing, National and Kapodistrian University of Athens, 11527 Athens, Greece; aglaiakat@nurs.uoa.gr; 3 Center for Health Services Management and Evaluation, Faculty of Nursing, National and Kapodistrian University of Athens, 11527 Athens, Greece; olympiak1982@hotmail.com; 4 Euro-Mediterranean Institute for Quality and Safety in Health Services, 10678 Athens, Greece; akatsapi@eiqsh.eu; 5 Palliative Care Unit Galilee, 19004 Spata, Greece; iprasini@galilee.gr

**Keywords:** workplace gaslighting, nurses, Gaslighting at Work Scale, job burnout, turnover intention

## Abstract

Νurses often experience abusive behavior, such as gaslighting, which has a negative impact on their mental health and leads them to quit their jobs. **Background/Objectives**: We evaluate the impact of workplace gaslighting on nurses’ job burnout and turnover intention. **Methods**: We conducted a cross-sectional study with a convenience sample of 410 nurses in Greece. We used the Gaslighting at Work Scale (GWS) to measure levels of workplace gaslighting in our sample. Also, we used the single-item burnout measure to measure job burnout and a six-point Likert scale to measure turnover intention. We constructed multivariable regression models to estimate the independent effect of workplace gaslighting on job burnout and turnover intention. **Results**: We found positive correlations between GWS and job burnout (r = 0.298, *p*-value < 0.01) and turnover intention (r = 0.385, *p*-value < 0.01). We found that workplace gaslighting was associated with job burnout in our sample. Our multivariable linear regression model identified a positive association between the score on the GWS (adjusted b = 0.653, 95% CI = 0.436 to 0.869, *p* < 0.001) and burnout. Similarly, we found that a higher score on the GWS was associated with a higher turnover intention (adjusted b = 0.616, 95% CI = 0.466 to 0.765, *p* < 0.001). **Conclusions**: This study findings indicate that nurses encounter gaslighting behaviors that adversely impact their job burnout and turnover intentions. Healthcare institutions are urged to implement policies that raise awareness about this conduct, facilitate avenues for staff to report it, and exhibit zero tolerance for abusive behaviors, including gaslighting.

## 1. Introduction

Nurses are the largest professional group in a hospital and deliver the greatest volume of patient care. The nature of their work and their working environment are highly demanding, as they must simultaneously address the psychosomatic requirements and the education of patients while assuring the quality, safety, and efficiency of the health services delivered. The support provided by supervisors is crucial for maintaining their workplace well-being and job retention. Nurses frequently endure abusive conduct from supervisors, and this adversely affects their mental health and compelling them to leave their positions [1,2].

Burnout has consistently been considered a phenomenon in the occupational lives of nurses. The overall pooled prevalence of burnout symptoms amongst nurses worldwide is estimated to be 11.23% [3]. Nonetheless, disparities exist throughout nations and various nursing specialties, with burnout prevalence fluctuating between 20% and 40% [4,5,6]. Nurses face burnout at a higher rate than any other healthcare professional group [7]. The most important model explaining the development of burnout, regardless of the work sector, is that of Job-Demands-Resources. Job demands denote the physical and mental exertion required from employees in their work context, whereas job resources encompass elements of the work environment that enable employees to fulfill their job responsibilities, mitigate the physical and psychological effects of job demands, and ultimately foster personal growth and development [8]. Significant job demands that lead to burnout in nurses include extended shifts, excessive workload, time constraints, and elevated job and psychological expectations [9,10]. The COVID-19 pandemic imposed arduous working circumstances on nurses, characterized by extended hours in quarantine zones, a high-risk workplace, and an elevated workload, all contributing to the onset of burnout among nursing professionals [11]. Concerning job resources, insufficient nurse staffing levels, employment at hospitals lacking suitable material and human resources, a deficient social climate or support, resilience, and restricted nurse involvement in hospital operations were predictors of nurse burnout [9,11,12]. Specifically, when nurses obtain support from their supervisor, their performance and affective organizational commitment is enhanced, whilst their intention to leave diminishes [13,14,15]. The ramifications of nurses’ burnout are multifaceted. Nurse burnout compromises the quality and safety of care, elevates patient dissatisfaction, diminishes nurse productivity, fosters quiet quitting, and heightens their turnover intention [16,17].

Due to the inability of healthcare organizations to provide adequate resources and support for nurses amidst significant work demands, nurses frequently resign from their positions or abandon the profession altogether. The proportion of nurses expressing their intention to quit their positions is significantly elevated, reaching 80% in the long run [18]. Nurses express a greater propensity to quit their jobs compared to other healthcare professions [19]. According to a study by the National Council of State Boards of Nursing (NCSBN), 100,000 nurses left the workforce during the pandemic in the USA and by 2027, almost 900,000, or almost one-fifth of 4.5 million total registered nurses, intend to leave the workforce [20]. The nurses’ expression of their intention to leave their jobs should prompt healthcare organizations to examine and improve the factors influencing this intention, as it is a robust predictor of actual turnover [21]. According to findings of an extensive study in the USA, 31.5% of nurses who reported leaving their job in 2017, stated burnout as a reason [22]. Poor work-related mental health outcomes are a predictive factor that elevates the probability of nurse turnover [23]. Elevated workplace demands, a poor work environment, and insufficient job resources may compel nurses to resign from their positions or exit the profession [23,24]. Moreover, adverse job experiences such as bullying, lateral aggression, and disrupted relationships can drive nurses to exit the field [23]. When nurse supervisors choose abusive leadership as a behavior trait, nurses’ turnover intention increases. When nurses work under a supportive leadership that inspires and motivates them, they report higher job contentment and lower intention to leave the nursing profession [25]. Studies on the role of negative leadership style in Greece are very limited. One study involving civil servants showed that destructive leadership negatively relates to leader–member exchange. A study of hospital employees investigated the relationship between Machiavellian leadership and emotional exhaustion, demonstrating their positive correlation [26,27].

Gaslighting is a form of psychological manipulation, which can be adopted and applied by those in positions of management to their subordinates. The term gaslighting first appeared in cinema in a film of the same name, in which the husband isolates his wife by convincing her that she is losing her grasp on reality. According to Stern “Gaslighting, is a type of emotional manipulation in which a gaslighter tries to convince you that you’re misremembering, misunderstanding, or misinterpreting your own behaviors or motivations, thus creating doubt in your mind that leaves you vulnerable or confused”. Various scientific disciplines, including psychology and sociology, have examined the phenomenon of gaslighting. Projective identification is akin to gaslighting, since the gaslighter externalizes their undesirable “psychic content” onto another individual, coercing them to accept and identify with that content. Another perspective posits that gaslighting involves a victim who must be inclined to alter their viewpoint to gain their spouse’s favor, alongside a partner who is resolute in their belief of being correct. This understanding of gaslighting has led to the development of a typology of perpetrators and the characteristics of individuals who are prone to victimization [28,29]. The sociological perspective posits that gaslighting is fundamentally linked to societal inequities, particularly gender, and is perpetrated within power-dominated interpersonal relationships. The sociological theory posits that gaslighting is significant when abusers exploit gender stereotypes and structural and institutional inequalities against victims to manipulate their realities [30].

Early reports viewed gaslighting as a conscious manipulative attempt to have a mentally healthy person committed to a psychiatric institution or hospital. Subsequent reports involved elderly people who were victims of gaslighting by workers or relatives in care homes or in their own homes. In each case, the ultimate purpose of the gaslighters was financial, emotional, or personal gain [31]. The gaslighter employs solely verbal communication and refrains from utilizing physical violence to attain his objectives. Gaslighting entails a perpetrator asserting that the victim is incapable of accurately understanding and forming valid views about reality; in other words, they are epistemically deficient. This is accomplished through several strategies, notably by making straightforward claims of epistemic incompetence, such as labeling the target as “mad” and asserting that the target is experiencing delusions [31]. Specifically, a gaslighter employs behavioral strategies, including lying and the questioning of perceptions, thoughts, feelings, or memories, to foster confusion and a sense of unreality. All these behaviors serve the overarching objective of inducing a sense of “craziness” in an individual, rendering them incapable of trusting their own thoughts and emotions. Another behavior is denial, which similarly aims to create a rift between a victim and their capacity to form judgments about reality. Denial strategies may involve the refusal to recognize facts, even when confronted with evidence. The gaslighter exhibits behavioral inconsistency, oscillating between egregious falsehoods and overt manipulation and more nuanced deceptions intermingled with occasional truths [29]. The gaslighter is driven by a desire for dominance and control, alongside personal insecurities, a compulsion to be correct, and a lust for power [31].

Gaslighting can emerge in relationships marked by inequality and power dynamics, and may be employed by individuals in positions of authority within an organization [30]. Individuals in positions of authority who engage in gaslighting behavior demonstrate considerable leadership deficiencies, opting to manipulate social interactions, spread disinformation, and cultivate confusion and doubt among others. Their conduct towards subordinates is accusatory, marked by Machiavellian tactics, intimidation, incessant criticism, and may even encompass the regulation of their subordinates’ personal lives [32]. When supervisors exhibit gaslighting behavior, it negatively affects employees’ affective organizational commitment and career entrenchment [33,34].

Despite the extensive discourse on gaslighting in the literature over the past decades, there is a paucity of studies examining its prevalence in workplace settings, particularly within the healthcare sector. This is likely attributable to the absence of a valid instrument for measuring workplace gaslighting. The first tool was developed in 2023 [35], whereas the second instrument utilized for this study was created in 2025 [36]. Two studies examined the influence of gaslighting on nursing personnel, both emphasizing its detrimental effects on nurses’ career commitment and agility [33,37]. To the best of our knowledge, this is the first study that investigates the impact of workplace gaslighting on nurses’ job burnout and turnover intention. In other words, the purpose of our study was to examine the association between workplace gaslighting, job burnout, and turnover intention in a sample of nurses. In particular, we considered the following hypotheses:

- There is an association between workplace gaslighting and job burnout. In particular, we hypothesized that higher levels of workplace gaslighting would be positively associated with higher levels of job burnout.
- There is an association between workplace gaslighting and turnover intention. In particular, we hypothesized that higher levels of workplace gaslighting would be positively associated with higher levels of turnover intention.

## 2. Materials and Methods

### 2.1. Study Design

We conducted a cross-sectional study with a convenience sample of 410 nurses in Greece. We collected our data during January 2025. We created an online version of the study questionnaire using Google forms. Then, we circulated our questionnaire in nurses’ groups on Facebook and Instagram. Nurses that have been working in clinical settings for at least one year could participate in our study. We applied the Strengthening the Reporting of Observational studies in Epidemiology (STROBE) guidelines in our study [38]. We conducted our study in accordance with the Declaration of Helsinki [39]. Moreover, the Ethics Committee of the Faculty of Nursing, National and Kapodistrian University of Athens approved our study protocol (approval number: 15, 9 December 2024).

### 2.2. Measurements

We measured the following demographic variables: gender (females or males), age (continuous variable), MSc/PhD diploma (no or yes), and work experience (continuous variable).

We used the Gaslighting at Work Scale (GWS) to measure levels of workplace gaslighting in our sample [36]. The GWS comprises 11 items, and answers are on a five-point Likert scale from never (1) to always (5). The GWS includes two factors: (a) “loss of self-trust” (five items) and (b) “abuse of power” (six items). Scores on the two factors range from 1 to 5. Higher scores indicate higher levels of workplace gaslighting. In our study, Cronbach’s alpha for the “loss of self-trust” and “abuse of power” factors were 0.900 and 0.908, respectively.

We used the single-item burnout measure to estimate job burnout in our sample. This item registers values from 0 to 10 [40]. Higher values indicate a higher level of job burnout. We used the valid Greek version of the scale [41].

We used a valid six-point Likert scale to measure turnover intention among our nurses [42]. We asked nurses “How often have you seriously considered leaving your current job?” and answers were on a scale from 1 (rarely) to 6 (extremely often). 

### 2.3. Statistical Analysis

We present categorical variables as numbers and percentages. Also, we use mean, standard deviation (SD), median, maximum value, and minimum value to present continuous variables. We considered workplace gaslighting as the independent variable and job burnout and turnover intention as the dependent variables. We considered our demographic variables as potential confounders. Since job burnout and turnover intention were continuous variables that followed normal distribution, we performed a linear regression analysis to examine the impact of workplace gaslighting on job burnout and turnover intention. We examined correlation between study scales with Pearson’s correlation coefficient. First, we performed a simple regression analysis, and then we constructed a final multivariable model by eliminating confounders to estimate the independent effect of workplace gaslighting on job burnout and turnover intention. We calculated variance inflation factors (VIFs) and tolerance values to assess multicollinearity in the multivariable models. A VIF greater than 5 and a tolerance value < 0.50 indicate multicollinearity between independent variables [43]. In the case of a linear regression, we present unadjusted and adjusted coefficients beta, 95% confidence intervals (CI), and *p*-values. Pearson’s correlation coefficient between age and work experience was very high (r = 0.941, *p*-value < 0.001). Similarly, Pearson’s correlation coefficient between the two factors (i.e., “loss of self-trust” and “abuse of power”) of the GWS was very high (r = 0.787, *p*-value < 0.001). Thus, to avoid multicollinearity issues in the multivariable linear regression models, we included the total score on the GWS instead of scores on the two separate factors. Moreover, we included work experience in the final multivariable models instead of including age and work experience simultaneously. We performed confirmatory factor analysis (CFA) to investigate common method bias in our study. We employed Harman’s single-factor test to examine common method bias. In that case, if the simple, one factor measurement model fits the data as well as the hypothesized model, then common method bias is present [44,45,46,47]. To examine how good the models are in the CFA, we calculated the following indices: chi-square/degree of freedom (x^2^/df), root mean square error of approximation (RMSEA), goodness of fit index (GFI), comparative fit index (CFI), and normed fit index (NFI). We used the following acceptable values: x^2^/df < 5, RMSEA < 0.10, GFI > 0.90, CFI > 0.90, and NFI > 0.90 [48,49,50]. *p*-values less than 0.05 were considered statistically significant. We used the IBM SPSS 28.0 (IBM Corp. Released 2021. IBM SPSS Statistics for Windows, Version 28.0. Armonk, NY, USA: IBM Corp) for the analysis. We used AMOS version 21 (Amos Development Corporation, 2018) to perform the CFA.

## 3. Results

### 3.1. Demographic Characteristics

The study population included 410 nurses. Most nurses were females (85.9%), while 14.9% were males. The mean age of our sample was 37.7 years (SD; 10.4) with a median age of 37 years. In our sample, 63.2% possessed a MSc/PhD diploma. Mean work experience was 13.74 years (SD; 10.2) with a median of 12 years. Demographic data of nurses are shown in Table 1.

### 3.2. Study Scales

The mean score on GWS was 2.6, while on factors of “loss of self-trust” and “abuse of power” the figures were 2.3 and 2.9, respectively. Levels of job burnout were high in our sample since the mean score for burnout was 6.8. Turnover intention was high among nurses since half of them (49.8%, n = 204) showed a high level of turnover intention. Descriptive statistics for the study scales are shown in Table 2.

Table 3 shows correlations between study scales. We found positive correlations between GWS and job burnout (r = 0.298, *p*-value < 0.01) and turnover intention (r = 0.385, *p*-value < 0.01). Similarly, we found positive correlations between scores on the factor of “loss of self-trust” and job burnout (r = 0.228, *p*-value < 0.01) and turnover intention (r = 0.323, *p*-value < 0.01). Moreover, we identified positive correlations between scores on the factor of “abuse of power” and job burnout (r = 0.323, *p*-value < 0.01) and turnover intention (r = 0.396, *p*-value < 0.01).

### 3.3. Impact of Workplace Gaslighting on Job Burnout

We found that workplace gaslighting was associated with job burnout in our sample. Our multivariable linear regression model identified a positive association between the score on the GWS (adjusted b = 0.653, 95% CI = 0.436 to 0.869, *p* < 0.001) and burnout. There were no multicollinearity issues since the tolerance value was 0.981, and the VIF was 1.020. Table 4 shows the results of the linear regression models with job burnout as the dependent variable.

### 3.4. Impact of Workplace Gaslighting on Turnover Intention

We found that workplace gaslighting is associated with nurses’ turnover intention. According to our multivariable linear regression model, a higher score on the GWS was associated with a higher turnover intention (adjusted b = 0.616, 95% CI = 0.466 to 0.765, *p* < 0.001). There were no multicollinearity issues since the tolerance value was 0.981 and the VIF was 1.020. Table 5 shows the results of the logistic regression models with turnover intention as the dependent variable.

### 3.5. Assessment of Common Methods Bias

The CFA showed an unacceptable fit for the simple, one factor measurement model since x^2^/df was 21.596, RMSEA was 0.224, GFI was 0.525, CFI was 0.600, and NFI was 0.590. On the other hand, the CFA for our hypothesized model with three factors (i.e., workplace gaslighting, job burnout, and turnover intention) suggested a very good fit to data since x^2^/df was 4.513, RMSEA was 0.090, GFI was 0.905, CFI was 0.938, and NFI was 0.953. Therefore, there was no common method bias since the simple, one factor measurement model did not fit the data as well as our hypothesized model.

## 4. Discussion

This study highlights the existence of gaslighting in the nursing workplace. According to our results, nurses experience moderate levels of gaslighting, which was found to have a positive effect on nurses’ job burnout and turnover intentions. This study is the first inquiry into the relationship between these variables, and it will reference the literature concerning the role of nursing leadership and these outcomes. Various leadership styles and organizational characteristics have been linked to nurses’ intention to remain in their positions. Transformational and authentic leadership styles, along with perceived organizational support, significantly decrease nurses’ turnover intention. When nurse leaders acknowledge employee contributions, implement mentoring, address individual employee needs, foster open communication, and encourage innovative behavior, creativity, and problem-solving, they diminish the likelihood of nurses resigning from their positions [24,51]. Authentic nurse leaders foster a pleasurable work atmosphere that promotes management openness, information sharing, the internalization of ethical principles, the support of autonomy, and constructive social interactions, hence increasing nurses’ desire to retain their positions. Ethical leadership also contributes to the retention of nurses in the workplace through relationship building, communication, decision-making, and reinforcement [52]. The proficient leadership of a nursing unit manager guarantees the quality and safety of care; in such a supportive managerial environment, there are fewer adverse patient events, improved reporting of nurse errors, and enhanced patient satisfaction and care quality [53]. 

Notwithstanding the aforementioned advantages of various leadership styles, nurses frequently encounter abusive conduct from their supervisors. Specifically, supervisors’ abusive conduct may encompass “silent treatment”, information withholding, rudeness, hostile eye contact, temper tantrums, explosive outbursts, intimidation, derogation, ridicule, and public humiliation, all of which significantly impact nurses’ decisions to leave the workplace [54]. Simultaneously, abusive supervision adversely impacts nurses’ occupational well-being, diminishing work engagement and exacerbating burnout [55]. Bullying of nurses, defined by recurrent negative and undesirable social behaviors, persists over an extended duration, with victims feeling unable to extricate themselves from the adverse circumstances or halt the negative interactions, and this has been linked to the onset of burnout and the intention of nurses to leave their positions [56]. The leadership style characterized by intemperate, egotistical, self-promoting, and humiliating behavior, typical of toxic leaders, has been established as a predictive factor for both nursing burnout and their decision to resign [57,58]. The quality and safety of nursing care are concurrently declining, as nurses indicate a rise in nurse-reported adverse events in departments characterized by toxic leadership behavior [59]. In addition to the abusive conduct of managers and its detrimental impact on employees, gaslighting behavior should be examined. While the research on gaslighting in the workplace is scarce, there exists a body of literature addressing its detrimental effects on employees. Supervisors who engage in gaslighting behavior adversely impact employee performance stability, career entrenchment, affective commitment, and agility [33,34,37,60].

The effects of nurses’ burnout and turnover are multifaceted and affect both patient care and health service organizations. Patients who are hospitalized in departments where nursing staff experience burnout report high rates of dissatisfaction and are also more likely to experience an adverse event [61]. Nurses’ turnover is also related to the quality of patient care, as well as creating staffing problems, and health care organizations are required to spend significant amounts of money to fill vacancies [62]. Health organization administrations should have a zero-tolerance policy against abusive behaviors, including gaslighting. Such behaviors are frequently challenging to expose, as the nurses who encounter them are suppressed. Health organizations predominantly focus on measuring patient satisfaction and documenting the quality of service, often overlooking the well-being of nurses and the determinants that affect it. Consequently, health care organization administrations should employ processes to evaluate supervisors’ leadership styles to accurately identify activities such as gaslighting.

Our study had several limitations. We conducted a cross-sectional study with a convenience sample that carries a high risk of self-selection and non-response bias. Thus, our sample could not be representative of the general population in Greece. For instance, the nurses’ educational level was very high in our study since 63.2% possessed a MSc/PhD diploma. Additionally, the majority of our participants were females (85.9%). Although the nurses’ population comprised mainly females, this high percentage in our study could also be attributed to the social media recruitment. More representative samples should be used in Greece to further validate our findings. Additional studies with samples from different countries and cultures should be conducted to further validate our findings. For instance, since working conditions are quite different among continents and countries, scholars should perform studies in several other countries to further examine our associations. Also, working conditions may be different among different clinical settings. For instance, scholars could examine the association between workplace gaslighting, turnover intention, and burnout among nurses working in the public or private sector. Additionally, further investigations could be made among nurses working in primary, secondary, or tertiary hospitals. Moreover, we used multivariable models to eliminate several confounders in our study, but several other variables can also act as confounders. Moreover, we cannot establish a causal relationship between workplace gaslighting, job burnout, and turnover intention since we conducted a cross-sectional study. Additionally, our multivariable models had low R^2^ values; therefore, workplace gaslighting explains a small percentage of the variability of job burnout and turnover intention. Thus, several other predictors may explain the variability in the of job burnout and turnover intention. Another limitation of our study was the inadequate control of confounders. Many socio-demographic and occupational factors may act as confounders in the association between workplace gaslighting and job burnout and turnover intention. Since we eliminated only a few confounders, scholars in future studies should further clarify this issue. We should note that sample recruitment via social media may introduce selection bias in our study. We posted our study questionnaire in nurses’ groups through social media where administrators allow access only to nurses. However, selection bias is still probable since we cannot guarantee that only nurses participate in these social media groups. Finally, we used valid instruments to measure workplace gaslighting, job burnout, and turnover intention in our sample. Moreover, the Greek version of the GWS is still a preprint and has not been published in a journal. Thus, information bias is probable in our study due to the self-reporting nature of these tools. Finally, although our hypothesized three-factor model fit the data better than a single-factor model, we should always recognize that common method bias is a potential issue in single-source cross-sectional studies such as our study.

## 5. Conclusions

Nurses are often victims of abusive behavior by their supervisors. One such form is gaslighting. Until recently, there was an inability to capture such behaviors as reliable tools to measure it were lacking. The findings of the present study show that nurses experience gaslighting to a moderate degree, which significantly influences the occurrence of burnout and nurses’ turnover intention. The well-being of nurses is fundamental to the efficient and effective functioning of health services and quality patient care. Also, nurses’ retention in their jobs ensures the functionality of services and, at the same time, the quality of care. Therefore, highlighting such work behaviors and zero tolerance by the administrations of healthcare organizations is the optimal solution against such abusive behaviors. Healthcare organizations must explicitly define gaslighting and incorporate it into their policies as intolerable conduct. Employees will readily identify such behavior and report it. Furthermore, the establishment by management of a culture that respects employees, provides psychological support for navigating daily challenges, and fosters collaboration and innovation will cultivate a healthy work environment, making it challenging for gaslighting practices to emerge. As this was a cross-sectional study using self-reporting tools, and no data recorded by health organizations were collected, further studies are needed to investigate the incidence of gaslighting in health organizations and its impact on nursing staff.

## Figures and Tables

**Table 1:** Demographic data of nurses (N = 410).

Characteristics	N	%
Gender		
Males	58	14.1
Females	352	85.9
Age (years) ^a^	37.7	10.4
MSc/PhD diploma		
No	151	36.8
Yes	259	63.2
Work experience (years) ^a^	13.7	10.2

^a^ Mean, standard deviation.

**Table 2:** Descriptive statistics for the study scales (N = 410).

Scale	Mean	Standard Deviation	Median	Minimum Value	Maximum Value
Gaslighting at Work Scale	2.6	1.0	2.5	1.0	5.0
Loss of self-trust	2.3	1.0	2.2	1.0	5.0
Abuse of power	2.9	1.0	2.8	1.0	5.0
Job burnout	6.8	2.2	7.0	0.0	10.0
Turnover intention	3.7	1.6	3.0	1.0	6.0

**Table 3:** Correlation matrix between study scales.

Scale	1	2	3	4	5
1 Gaslighting at Work Scale		0.930 *	0.959 *	0.298 *	0.385 *
2 Loss of self-trust			0.787 *	0.228 *	0.323 *
3 Abuse of power				0.323 *	0.396 *
4 Job burnout					0.531 *
5 Turnover intention					

Values express Pearson’s correlation coefficient. * *p*-value < 0.01.

**Table 4:** Linear regression models with job burnout as the dependent variable (N = 410).

	Univariate Models			Multivariable Model ^a^		
	Unadjusted Coefficient Beta	95% CI for Beta	*p*-Value	Adjusted Coefficient Beta	95% CI for Beta	*p*-Value
Workplace gaslighting	0.700	0.481 to 0.918	<0.001	0.653	0.436 to 0.869	<0.001

^a^ R^2^ for the multivariable model = 13.5%, *p*-value for ANOVA < 0.001. CI: confidence interval.

**Table 5:** Linear regression models with turnover intention as the dependent variable (N = 410).

	Univariate models			Multivariable Model ^a^		
	Unadjusted Coefficient Beta	95% CI for Beta	*p*-Value	Adjusted Coefficient Beta	95% CI for Beta	*p*-Value
Workplace gaslighting	0.637	0.488 to 0.786	<0.001	0.616	0.466 to 0.765	<0.001

^a^ R^2^ for the multivariable model = 12.3%. CI: confidence interval.

## Data Availability

The original data presented in the study are openly available in FigShare at https://doi.org/10.6084/m9.figshare.28868615.

## References

[1] Simard K., Parent-Lamarche A. (2022). Abusive Leadership, Psychological Well-Being, and Intention to Quit during the COVID-19 Pandemic: A Moderated Mediation Analysis among Quebec’s Healthcare System Workers. Int. Arch. Occup. Environ. Health.

[2] Broetje S., Jenny G.J., Bauer G.F. (2020). The Key Job Demands and Resources of Nursing Staff: An Integrative Review of Reviews. Front. Psychol..

[3] Woo T., Ho R., Tang A., Tam W. (2020). Global Prevalence of Burnout Symptoms among Nurses: A Systematic Review and Meta-Analysis. J. Psychiatr. Res..

[4] Ramírez-Elvira S., Romero-Béjar J.L., Suleiman-Martos N., Gómez-Urquiza J.L., Monsalve-Reyes C., Cañadas-De la Fuente G.A., Albendín-García L. (2021). Prevalence, Risk Factors and Burnout Levels in Intensive Care Unit Nurses: A Systematic Review and Meta-Analysis. Int. J. Environ. Res. Public Health.

[5] López-López I.M., Gómez-Urquiza J.L., Cañadas G.R., De la Fuente E.I., Albendín-García L., Cañadas-De la Fuente G.A. (2019). Prevalence of Burnout in Mental Health Nurses and Related Factors: A Systematic Review and Meta-Analysis. Int. J. Ment. Health Nurs..

[6] Gómez-Urquiza J.L., De la Fuente-Solana E.I., Albendín-García L., Vargas-Pecino C., Ortega-Campos E.M., Cañadas-De la Fuente G.A. (2017). Prevalence of Burnout Syndrome in Emergency Nurses: A Meta-Analysis. Crit. Care Nurse.

[7] Galanis P., Moisoglou I., Katsiroumpa A., Vraka I., Siskou O., Konstantakopoulou O., Meimeti E., Kaitelidou D. (2023). Increased Job Burnout and Reduced Job Satisfaction for Nurses Compared to Other Healthcare Workers after the COVID-19 Pandemic. Nurs. Rep..

[8] Demerouti E., Bakker A.B., Nachreiner F., Schaufeli W.B. (2001). The Job Demands-Resources Model of Burnout. J. Appl. Psychol..

[9] Dall’Ora C., Ball J., Reinius M., Griffiths P. (2020). Burnout in Nursing: A Theoretical Review. Hum. Resour. Health.

[10] Galanis P., Moisoglou I., Katsiroumpa A., Gallos P., Kalogeropoulou M., Meimeti E., Vraka I., Galanis P., Moisoglou I., Katsiroumpa A. (2025). Workload Increases Nurses’ Quiet Quitting, Turnover Intention, and Job Burnout: Evidence from Greece. AIMSPH.

[11] Galanis P., Vraka I., Fragkou D., Bilali A., Kaitelidou D. (2021). Nurses’ Burnout and Associated Risk Factors during the COVID-19 Pandemic: A Systematic Review and Meta-Analysis. J. Adv. Nurs..

[12] Moisoglou I., Katsiroumpa A., Malliarou M., Papathanasiou I.V., Gallos P., Galanis P. (2024). Social Support and Resilience Are Protective Factors against COVID-19 Pandemic Burnout and Job Burnout among Nurses in the Post-COVID-19 Era. Healthcare.

[13] Chami-Malaeb R. (2021). Relationship of Perceived Supervisor Support, Self-Efficacy and Turnover Intention, the Mediating Role of Burnout. Pers. Rev..

[14] Ravangard R., Yasami S., Shokrpour N., Sajjadnia Z., Farhadi P. (2015). The Effects of Supervisors’ Support and Mediating Factors on the Nurses’ Job Performance Using Structural Equation Modeling: A Case Study. Health Care Manag..

[15] Orgambídez A., Almeida H. (2020). Supervisor Support and Affective Organizational Commitment: The Mediator Role of Work Engagement. West. J. Nurs. Res..

[16] Jun J., Ojemeni M.M., Kalamani R., Tong J., Crecelius M.L. (2021). Relationship between Nurse Burnout, Patient and Organizational Outcomes: Systematic Review. Int. J. Nurs. Stud..

[17] Galanis P., Katsiroumpa A., Vraka I., Siskou O., Konstantakopoulou O., Katsoulas T., Moisoglou I., Gallos P., Kaitelidou D. (2023). The Influence of Job Burnout on Quiet Quitting among Nurses: The Mediating Effect of Job Satisfaction. Res. Sq..

[18] Labrague L., De Los Santos J.A., Falguera C., Nwafor C., Galabay J., Rosales R., Firmo C. (2020). Predictors of Nurses’ Turnover Intention at One and Five Years’ Time. Int. Nurs. Rev..

[19] Rotenstein L.S., Brown R., Sinsky C., Linzer M. (2023). The Association of Work Overload with Burnout and Intent to Leave the Job Across the Healthcare Workforce During COVID-19. J. Gen. Intern. Med..

[20] NCSBN Research Projects Significant Nursing Workforce Shortages and Crisis. https://www.ncsbn.org/news/ncsbn-research-projects-significant-nursing-workforce-shortages-and-crisis.

[21] Ki J., Choi-Kwon S. (2022). Health Problems, Turnover Intention, and Actual Turnover among Shift Work Female Nurses: Analyzing Data from a Prospective Longitudinal Study. PLoS ONE.

[22] Shah M.K., Gandrakota N., Cimiotti J.P., Ghose N., Moore M., Ali M.K. (2021). Prevalence of and Factors Associated With Nurse Burnout in the US. JAMA Netw. Open.

[23] Leep-Lazar K., Ma C., Stimpfel A.W. (2025). Factors Associated with Intent to Leave the Nursing Profession in the United States: An Integrative Review. Res. Nurs. Health.

[24] Galanis P., Moisoglou I., Papathanasiou I.V., Malliarou M., Katsiroumpa A., Vraka I., Siskou O., Konstantakopoulou O., Kaitelidou D. (2024). Association between Organizational Support and Turnover Intention in Nurses: A Systematic Review and Meta-Analysis. Healthcare.

[25] Labrague L.J., Nwafor C.E., Tsaras K. (2020). Influence of Toxic and Transformational Leadership Practices on Nurses’ Job Satisfaction, Job Stress, Absenteeism and Turnover Intention: A Cross-Sectional Study. J. Nurs. Manag..

[26] Gkorezis P., Petridou E., Krouklidou T. (2015). The Detrimental Effect of Machiavellian Leadership on Employees’ Emotional Exhaustion: Organizational Cynicism as a Mediator. Eur. J. Psychol..

[27] Bellou V., Dimou M. (2022). The Impact of Destructive Leadership on Public Servants’ Performance: The Mediating Role of Leader-Member Exchange, Perceived Organizational Support and Job Satisfaction. Int. J. Public. Adm..

[28] Stern R. (2007). The Gaslight Effect: How to Spot and Survive the Hidden Manipulation Others Use to Control Your Life.

[29] Darke L., Paterson H., van Golde C. (2025). Illuminating Gaslighting: A Comprehensive Interdisciplinary Review of Gaslighting Literature. J. Fam. Viol..

[30] Sweet P.L. (2019). The Sociology of Gaslighting. Am. Sociol. Rev..

[31] Klein W., Wood S., Bartz J. (2023). A Historical Review of Gaslighting: Tracing Changing Conceptualizations Within Psychiatry and Psychology. OSF.

[32] Yasini A., Adinehvand M., Kabiri Yeganeh M. (2025). Understanding the Phenomenon of Gaslighting in Organizational Relationships. Public Organ. Manag..

[33] Atta M.H.R., Waheed Elzohairy N., Abd Elaleem A.E.D.M.H., Othman A.A., Hamzaa H.G., El-Sayed A.A.I., Zoromba M.A. (2025). Comprehending the Disruptive Influence of Workplace Gaslighting Behaviours and Mobbing on Nurses’ Career Entrenchment: A Multi-Centre Inquiry. J. Adv. Nurs..

[34] Fulcher C., Ashkanasy N.M. (2023). Supervisory Gaslighting and Its Effects on Employee Affective Commitment. Emotions During Times of Disruption.

[35] Kukreja P., Pandey J. (2023). Workplace Gaslighting: Conceptualization, Development, and Validation of a Scale. Front. Psychol..

[36] Katsiroumpa A., Moisoglou I., Konstantakopoulou O., Tsiachri M., Kolisiati A., Galanis P. (2025). The Gaslighting at Work Scale: Development and Initial Validation. Res. Sq..

[37] El-Sayed A.A.I., Alsenany S.A., Atta M.H.R., Othman A.A., Asal M.G.R. (2025). Navigating Toxicity: Investigating the Interplay Between Workplace Gaslighting, Workaholism, and Agility Among Nurses. Nurs. Inq..

[38] Von Elm E., Altman D.G., Egger M., Pocock S.J., Gøtzsche P.C., Vandenbroucke J.P. (2007). The Strengthening the Reporting of Observational Studies in Epidemiology (STROBE) Statement: Guidelines for Reporting Observational Studies. Lancet.

[39] World Medical Association (2013). World Medical Association Declaration of Helsinki: Ethical Principles for Medical Research Involving Human Subjects. JAMA.

[40] Hansen V., Pit S. (2016). The Single Item Burnout Measure Is a Psychometrically Sound Screening Tool for Occupational Burnout. Health Scope.

[41] Galanis P., Katsiroumpa A., Vraka I., Siskou O., Konstantakopoulou O., Katsoulas T., Gallos P., Kaitelidou D. (2024). The Single Item Burnout Measure Is a Reliable and Valid Tool to Measure Occupational Burnout. Arch. Hell. Med./Arheia Ellenikes Iatr..

[42] Spector P.E., Dwyer D.J., Jex S.M. (1988). Relation of Job Stressors to Affective, Health, and Performance Outcomes: A Comparison of Multiple Data Sources. J. Appl. Psychol..

[43] Fox J., Kempf-Leonard K. (2005). Linear Models, Problems. Encyclopedia of Social Measurement.

[44] Kock F., Berbekova A., Assaf A.G. (2021). Understanding and Managing the Threat of Common Method Bias: Detection, Prevention and Control. Tour. Manag..

[45] Fuller C.M., Simmering M.J., Atinc G., Atinc Y., Babin B.J. (2016). Common Methods Variance Detection in Business Research. J. Bus. Res..

[46] Korsgaard M.A., Roberson L. (1995). Procedural Justice in Performance Evaluation: The Role of Instrumental and Non-Instrumental Voice in Performance Appraisal Discussions. J. Manag..

[47] Podsakoff P.M., MacKenzie S.B., Lee J.-Y., Podsakoff N.P. (2003). Common Method Biases in Behavioral Research: A Critical Review of the Literature and Recommended Remedies. J. Appl. Psychol..

[48] Baumgartner H., Homburg C. (1996). Applications of Structural Equation Modeling in Marketing and Consumer Research: A Review. Int. J. Res. Mark..

[49] Hu L., Bentler P.M. (1998). Fit Indices in Covariance Structure Modeling: Sensitivity to Underparameterized Model Misspecification. Psychol. Methods.

[50] Brown T.A. (2015). Confirmatory Factor Analysis for Applied Research.

[51] Pattali S., Sankar J.P., Al Qahtani H., Menon N., Faizal S. (2024). Effect of Leadership Styles on Turnover Intention among Staff Nurses in Private Hospitals: The Moderating Effect of Perceived Organizational Support. BMC Health Serv. Res..

[52] Lambert J.R., Brown L.W., Lambert T.A., Torres Nava C. (2024). The Effect of Ethical Leadership on Nurse Bullying, Burnout, and Turnover Intentions. J. Nurs. Manag..

[53] Lee S.E., Hyunjie L., Sang S. (2023). Nurse Managers’ Leadership, Patient Safety, and Quality of Care: A Systematic Review. West. J. Nurs. Res..

[54] Lyu D., Ji L., Zheng Q., Yu B., Fan Y. (2019). Abusive Supervision and Turnover Intention: Mediating Effects of Psychological Empowerment of Nurses. Int. J. Nurs. Sci..

[55] Labrague L.J. (2024). Abusive Supervision and Its Relationship with Nursing Workforce and Patient Safety Outcomes: A Systematic Review. West. J. Nurs. Res..

[56] Galanis P., Moisoglou I., Katsiroumpa A., Sourtzi P. (2024). Impact of Workplace Bullying on Job Burnout and Turnover Intention among Nursing Staff in Greece: Evidence after the COVID-19 Pandemic. AIMS Public Health.

[57] Labrague L.J. (2023). Toxic Leadership and Its Relationship with Outcomes on the Nursing Workforce and Patient Safety: A Systematic Review. Leadersh. Health Serv..

[58] Nonehkaran E.A., Mozaffari N., Iranpour S., Soola A.H. (2023). Identifying the Predictors of Turnover Intention Based on Nurse Managers’ Toxic Leadership Behaviors among Nurses in Iran: A Cross-Sectional Correlational Study. BMC Health Serv. Res..

[59] Labrague L.J. (2021). Influence of Nurse Managers’ Toxic Leadership Behaviours on Nurse-Reported Adverse Events and Quality of Care. J. Nurs. Manag..

[60] Tongo N.I., Oloke E., Aransiola I.J., Eke A., Olowo S. (2023). Gaslighting And Employees’ Sustainable Performance: An Empirical Study of Nestle Nigeria PLC. Public. Adm. Reg. Stud..

[61] Li L.Z., Yang P., Singer S.J., Pfeffer J., Mathur M.B., Shanafelt T. (2024). Nurse Burnout and Patient Safety, Satisfaction, and Quality of Care: A Systematic Review and Meta-Analysis. JAMA Netw. Open.

[62] Bae S.-H. (2022). Noneconomic and Economic Impacts of Nurse Turnover in Hospitals: A Systematic Review. Int. Nurs. Rev..

